# Exploring the causal relationship between interleukin-6 or C reactive protein and malignant melanoma using a two-sample Mendelian randomization approach

**DOI:** 10.3389/fonc.2024.1375362

**Published:** 2024-06-17

**Authors:** Quan Jun Wang, Wei Zheng, Sun Feng Pan

**Affiliations:** ^1^ Zhejiang Chinese Medical University, Hangzhou, Zhejiang, China; ^2^ Department of Burns and Plastic Surgery, Affiliated Hospital of Jiangsu University, Zhenjiang, Jiangsu, China; ^3^ Department of Burns and Plastic Surgery, Jiaxing Hospital of Traditional Chinese Medicine, Zhenjiang, Jiaxing, China

**Keywords:** Mendelian randomization, malignant melanoma, C-reactive protein, interleukin-6, causal association

## Abstract

The goal was to explore the effect of interleukin-6 (IL6) and C reactive protein (CRP) on malignant melanoma (MM) using two-sample Mendelian randomization.

**Methods:**

Data for this study were obtained from the IEU Open GWAS project website for genome-wide association study data (GWAS) on interleukin-6, C reactive protein levels and malignant melanoma. Inverse variance weighted (IVW) method was mainly used and supplemented with MR-Egger regression and weighted median. Finally, horizontal multivariate validity and heterogeneity tests were performed to assess the stability and reliability of the results.

**Results:**

The results of univariate two-sample MR analyses showed no significant effect of CRP on MM: inverse variance weighting method (OR=0.999, 95% CI: 0.998–1.001, P=0.343), MR-Egger regression (OR= 1.000, 95% CI: 0.998–1.001, P= 0.180), and weighted median method (OR= 0.999, 95% CI: 0.997 to 1.000, P= 0.583), and weighted model (OR= 0.999, 95% CI: 0.998 to 1.001, P= 0.328). Also,IL-6 had no significant effect on MM: inverse variance weighting method (OR= 1.001, 95% CI: 0.999 to 1.002, P=0.461), MR-Egger regression (OR= 1.000, 95% CI: 0.997 to 1.004, P= 0.910), weighted median method (OR= 1.000, 95% CI: 0.998 to 1.002, P= 0.749), and weighted mode (OR= 1.000, 95% CI: 0.998 to 1.002, P= 0.820).

**Conclusion:**

There was no causal relationship between C-reactive protein and IL-6 on the risk of malignant melanoma.

## 1 Introduction

Malignant melanoma is an aggressive tumor differentiated from melanocytes (1). Epidemiology studies indicated that the incidences of MM are increasing significantly worldwide. The lethality rate reaches high up to 90% (2, 3). It is estimated that 1 in 63 Americans have melanoma (4, 5). MM generally implicates skin tissue, eyes and mucous membranes. Surgery is the main treatment method (2, 6), the margins of resection were determined based on the depth of the tumor. Other treatments such as targeted therapies and immunotherapy have changed the paradigm for advanced melanoma significantly with the development of therapeutic options. However, the prognosis of MM remains less favorable (7). Early diagnosis and timely treatment of MM increases the 5-year overall survival up to 95%. Previously only 5% of MM patients are long-term survivors after metastasis (7, 8). However, in stage IV metastatic melanoma, immunotherapy had excellent results, reaching 50% survival 5 years after treatment with the nivo+ipi combination, but these patients may be at high risk for new exacerbations or drug toxicity while continuing co-administration (9). Therefore, how to effectively diagnose and prevent MM at early stage is crucial to the prognosis of patients.

IL-6 is a pleiotropic cytokine that is almost universally expressed in stromal and immune cells (10). It not only involved in immune response, but also in basic processes such as inflammation, hematopoiesis, bone metabolism and embryonic development (11). The producing process of IL-6 is regulated by a variety of signals in normal cells such as tumor necrosis factor-a (TNF-a), interleukin-1, interferon-b, DNA viruses and RNA viruses (12). IL-6 plays an important role in the pathogenesis and progression of malignant tumors by promoting tumor growth through inhibiting apoptosis and induces tumor angiogenesis (13, 14). It can induce hepatocytes to generate CRP (15). The latter is a member of the pentapeptide protein family secreted by liver in response to a variety of inflammatory cytokines. CRP binds to lysophosphatidylcholine on the surfaces of dead or dying eukaryotic cells and bacteria, and then activates complement via C1q (16). Levels of CRP in serum are markedly increasing in acute inflammatory responses and have little difference in chronic inflammatory responses (17). CRP greater than 10 mg/L is usually associated with infections, certain inflammatory diseases or malignancies (18). A study has shown that high levels of CRP induce an immunosuppressive milieu in melanoma and supports blocking CRP as a therapeutic strategy to augment cancer immune checkpoint therapies (19). Several studies have suggested that IL-6 and CRP may be associated with diagnosis and prognosis of melanoma (12, 17, 20–22). However, it is difficult for traditional observational studies to avoid the interference of potential confounders and reverse causation, which may produce some bias in the results. Therefore, the effects of IL-6 or CRP on MM remain somewhat controversial.

## 2 Materials and methods

### 2.1 Research and design

**Figure 1:**
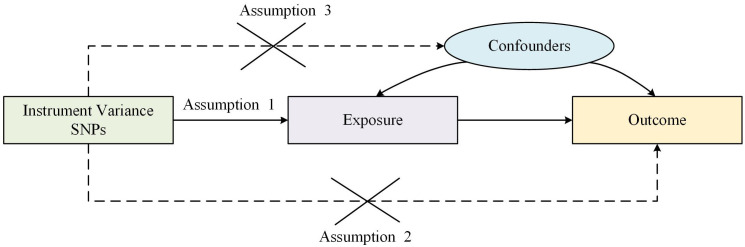
Three assumptions of Mendelian randomization.

Traditional observational epidemiologic studies face many challenges in discovering disease etiology and inferring causality, such as reverse causation, potential confounders and secondary exposure factors. It is difficult to explore the causal relationship between C-reactive protein and MM in observational and retrospective cohort studies due to the presence of treatment or other confounding factors beyond our control. Mendelian randomization (MR) is a statistical method which uses genetic variants as proxy markers for risk factors for instrumental variable analysis and is based on whole genome sequencing data used to expose causal relationships (23). Thereby, bias in traditional observational studies is circumvented as well as reduced exposure to confounding and reverse causation. Two-sample MR must satisfy the following three core assumptions: (1) IVs is required to be strongly associated with exposure; (2) IVs are not associated with confounders affecting “exposure-endpoints”; (3) IVs affect endpoints through exposure only but not through other pathways as shown in **Figure 1**.

### 2.2 Data sources

The data for this study were acquired from the GWAS of IL-6, CRP and MM from the website, gwas.mrcieu.ac.uk, and standardized to the European population database. The sample size of IL-6 database is 21,758 cases and the number of SNPs is 11,782,139; the sample size of CRP database is 353,466 cases and the number of SNPs is 19,057,467; the sample size of MM database is 462,933 cases and the number of SNPs is 9,851,867, as shown in **Table 1**.

**Table 1:** Summary of the GAWS included in this two sample MR study.

	GWAS ID	Sample size	Number of SNPS	Population	Year
Malignant Melanoma	ukb-b-12915	462933	9851867	European	2018
Interleukin-6 levels	ebi-a-GCST90012005	21758	33067605	European	2020
C-reactive protein	ebi-a-GCST90018950	353466	34594039	European	2021

### 2.3 Selection of SNPs

To satisfy assumption 1-IVs are required closely associated with exposure-P<5 × 10^-8^ is generally used as the screening condition, SNPs which are significantly associated with exposure were extracted from the GWAS database as the IVs. The linkage disequilibrium is removed (r^2^ ≤ 0.001; genetic distance<10,000 kilobases) to ensure independence of instrumental variable. Major and minor allele data is extracted for each SNP as well as allele frequencies, β coefficients, standard error (SE) of β coefficients and p-values of relevant associations (24). The F-value of IVs is calculated in the end and the corresponding IVs are included in the study to avoid bias caused by weak IVs if F>10.

In the study of CRP and MM, screening was performed according to the conditions indicated above and a total of 135 SNPs were included for MR analysis of CRP and MM at last. In the study of IL-6 and MM, P<5 × 10^-6^ as the screening condition was merged with the MM data set due to the small number of SNPs and incorporated eight SNPs for MR analysis of IL-6 and MM, as shown in **Table 2**.

**Table 2:** Information of the selected SNPs analyzed by MR.

SNPs	Exposures	Outcome	CHR	EA	OA	EAF	β	SE	P
rs10175899	CRP	melanoma	2	A	T	0.688408	0.0154	0.0023	3.31E-11
rs1037170	CRP	melanoma	17	T	C	0.682071	0.0251	0.0024	1.61E-26
rs10831676	CRP	melanoma	11	C	A	0.52331	-0.0122	0.0022	4.52E-08
rs11039148	CRP	melanoma	11	A	T	0.154873	-0.0333	0.0033	1.56E-23
rs11078597	CRP	melanoma	17	C	T	0.189012	-0.0187	0.0027	6.37E-12
rs11230768	CRP	melanoma	11	A	G	0.268401	0.0148	0.0025	5.54E-09
rs112875651	CRP	melanoma	8	A	G	0.350504	-0.0201	0.0023	3.89E-18
rs113040517	CRP	melanoma	19	C	G	0.763448	0.0184	0.0029	1.66E-10
rs11577023	CRP	melanoma	1	C	T	0.309517	-0.016	0.0026	1.02E-09
rs11708067	CRP	melanoma	3	G	A	0.198778	0.0163	0.0028	5.87E-09
rs11738559	CRP	melanoma	5	T	C	0.171287	0.0184	0.0029	2.25E-10
rs12132412	CRP	melanoma	1	G	A	0.351357	0.0145	0.0023	4.91E-10
rs1490384	CRP	melanoma	6	T	C	0.592164	-0.0307	0.0024	1.76E-37
rs1545536	CRP	melanoma	8	T	C	0.256588	-0.0158	0.0025	2.67E-10
rs1546721	CRP	melanoma	6	C	T	0.393537	-0.0122	0.0022	2.99E-08
rs17088032	CRP	melanoma	4	A	T	0.165794	0.0179	0.0032	3.10E-08
rs17138478	CRP	melanoma	17	A	C	0.144262	0.0318	0.003	2.14E-25
rs17652767	CRP	melanoma	15	A	G	0.115538	-0.0237	0.0033	1.37E-12
rs178795	CRP	melanoma	17	A	G	0.438631	0.0139	0.0023	2.53E-09
rs687339	CRP	melanoma	3	T	C	0.781408	-0.0229	0.0026	1.29E-18
rs6920220	CRP	melanoma	6	A	G	0.180228	0.0203	0.0029	1.76E-12
rs7012637	CRP	melanoma	8	A	G	0.554123	0.0451	0.0023	1.01E-82
rs7673508	CRP	melanoma	4	T	C	0.409241	-0.0123	0.0022	4.16E-08
rs8060025	CRP	melanoma	16	G	T	0.545205	-0.0156	0.0022	3.13E-12
rs832578	CRP	melanoma	5	T	C	0.827135	0.0187	0.0032	4.40E-09
rs9366639	CRP	melanoma	6	G	C	0.155422	-0.0281	0.003	7.40E-21
rs939584	CRP	melanoma	2	T	C	0.841583	0.0167	0.0029	1.45E-08
rs9521499	CRP	melanoma	13	C	T	0.462515	0.0128	0.0022	6.44E-09
rs1444691	IL-6	melanoma	2	G	A	0.402	-0.0573	0.0122	2.69E-06
rs1530088	IL-6	melanoma	16	T	C	0.7709	0.068	0.0147	3.95E-06
rs2228145	IL-6	melanoma	1	C	A	0.3786	0.1747	0.0124	3.34E-45
rs2288477	IL-6	melanoma	19	G	A	0.3368	-0.0617	0.0133	3.74E-06
rs2651097	IL-6	melanoma	19	A	G	0.2506	-0.071	0.0155	4.52E-06
rs4802241	IL-6	melanoma	19	C	A	0.1771	-0.0791	0.0166	1.85E-06
rs4959106	IL-6	melanoma	6	C	T	0.4615	0.0823	0.0138	2.37E-09
rs7448500	IL-6	melanoma	5	G	C	0.694	0.0631	0.0128	8.68E-07

SNPs, Single nucleotide polymorphisms; CHR, Chromosome; EA, Effect allele; OA, Other allele; POS;EAF, Effect allele frequency; β, Effect allele value; SE, Standard error.

### 2.4 MR analysis

In this study, four analytical methods is used to assess the causal relationship between exposure factors and outcomes, including four methods: IVW, MR-Egger regression, weighted median method and weighted mode method (25). IVW was the predominant analytical method. The results of it are the most reliable of all when the heterogeneity and horizontal polytomous effects are absent (26). MR-Egger regression was performed under the InSIDE assumption. Thus, assessing the presence of multinomial using the intercept term is allowed. When the intercept term was 0, it indicated the absence of horizontal multinomial and led to the inference that the results of the MR-Egger regression were consistent with IVW (27). Weighted median method provided a more accurate estimation of causality when the instrumental variable was more than 50% of invalidation. Weighted mode estimation method can detect causality better and with less deviation than MR-Egger regression method When the InSIDE assumption was not satisfied (28).

## 3 Results

### 3.1 Results of MR analysis

**Figure 2:**
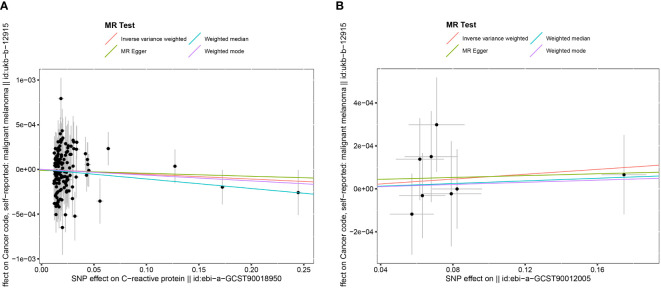
The Scatter plot of the association between C reactive protein and malignant melanoma. **(A)** The Scatter plot of the association between interleukin-6 and malignant melanoma **(B)**.

In the association study between CRP and MM, the results of IVW method indicated that CRP had no significant effect on MM (OR= 0.999, 95% CI: 0.998–1.001, P= 0.343), and MR-Egger regression (OR= 1.000, 95% CI: 0.998–1.001, P= 0.180). The results of the other 2 methods (weighted median method and weighted mode) were on the same trends as the IVW results as shown in **Figure 2A**.

In the association study between IL-6 and MM, the results of IVW method showed that IL-6 had no significant effect on MM (OR= 1.001, 95% CI: 0.999–1.002, P=0.461), and the MR-Egger regression (OR= 1.000, 95% CI: 0.997–1.004, P=0.910). The results of the other 2 methods (weighted median method and weighted mode) were in the same trends as the IVW results as shown in **Figure 2B**.

### 3.2 Sensitivity analysis results

The P-value of Cochran Q was used to infer the heterogeneity of MR results in this study (27). If P < 0.05, it indicates that there were no heterogeneity in the included instrumental variables. In the association study between CRP and MM, Cochran Q resulted in the absence of heterogeneity in both IVW analysis (Cochran Q=169.1, P=0.113) and MR-Egger regression analysis (Cochran Q=169.1, P=0.103). The results of the MR regression analysis showed that horizontal multiplicity had no significant influence (intercept=9×10–7, P=0.971).

**Figure 3:**
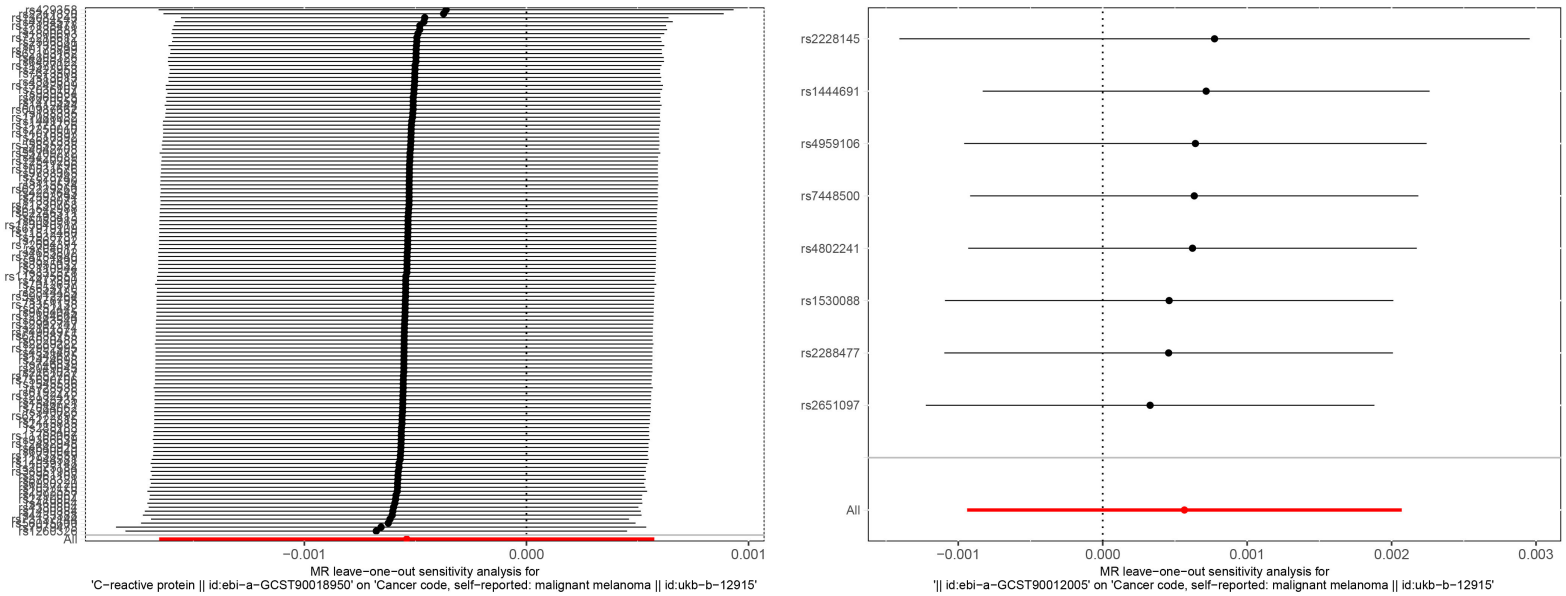
“Leave-one-out” sensitivity analysis of the causal effect of CRP and melanoma (left). “Leave-one-out” sensitivity analysis of the causal effect of IL-6 and melanoma (right).

**Figure 4:**
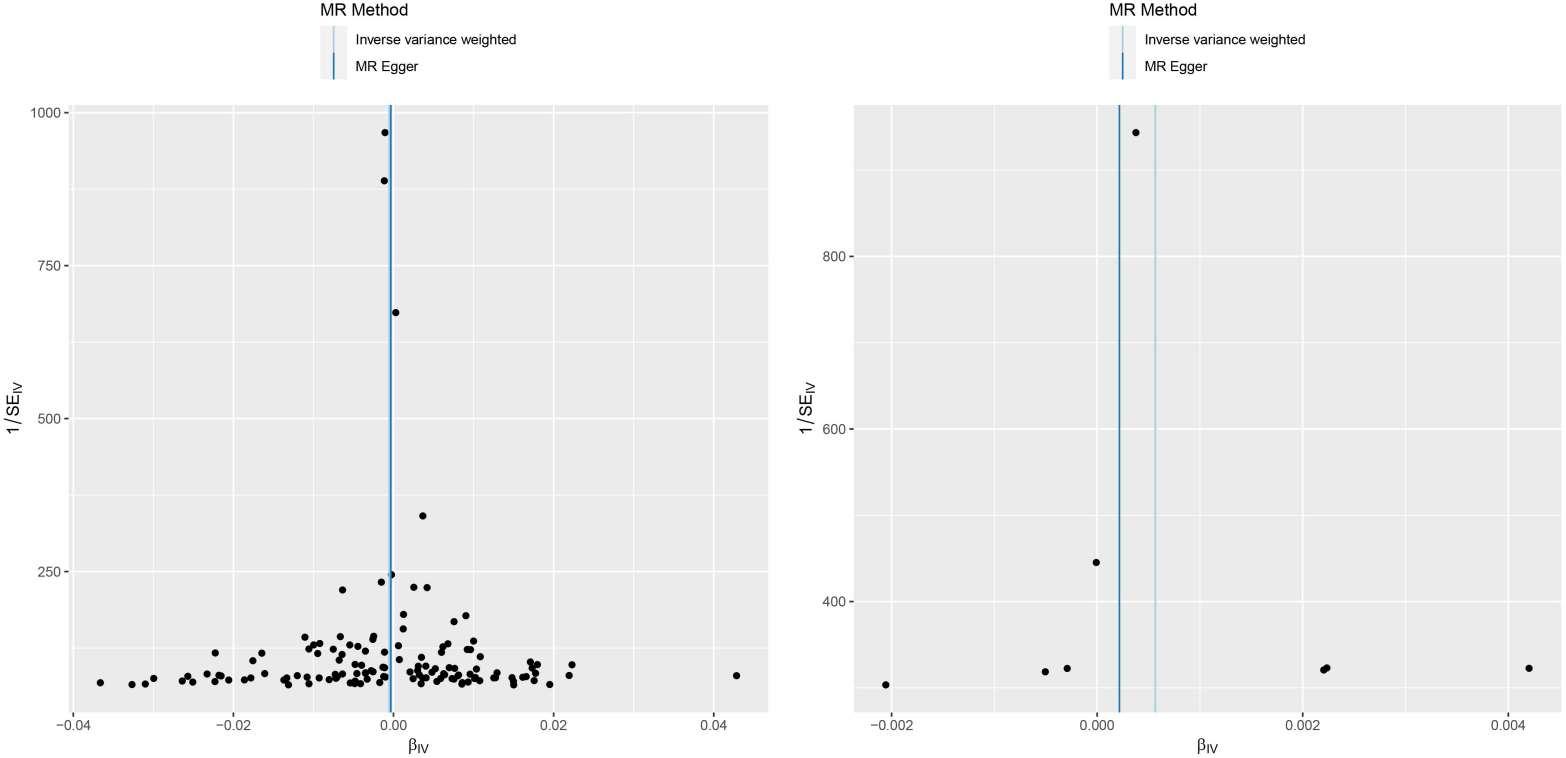
A funnel plot of the association between C reactive protein and malignant melanoma (left). A funnel plot of the association between interleukin-6 and malignant melanoma (right).

In the association study between IL-6 and MM, the results of Cochran Q for IVW analysis (Cochran Q=2.866, P=0.897) and MR-Egger regression analysis (Cochran Q=2.822, P=0.831) were not heterogeneous. The results of the MR regression analysis showed that horizontal pleiotropy had no significant effect on the results of MR analysis (intercept=3.6×10–5, P=0.842) as well. The results of “leave-one-out” method suggested that excluding SNPs separately did not significantly affect the causal associations which indicated the reliability of the MR results as shown in **Figure 3**. The symmetry between the two sides of the distribution of the funnel plot also indicated that the causal associations were less affected by the potential bias as shown in **Figure 4**.

## 4 Discussion

Exploring the causal relationship between IL-6 or CRP and MM is vital for the early diagnosis and prognosis of the disease. No current study has demonstrated the underlying mechanisms clearly between IL-6 or CRP and MM.

The relationship between IL-6 and MM has been discussed for decades. However, the underlying mechanisms remain unclear. A link between inflammation and cancer has long been suspected and it has been hypothesized that at least 15% of cancers are developed from chronic inflammation or infection (29). In clinical practice, physicians always seek a balance between protecting their own immunity and ensuring a good tumor response, prolonged use of immunosuppressive agents or use at ICI initiation may impede antitumor immune response, but Inhibitors of IL-6 can synergize with ICIs on tumor response and also prevent severe immune-related adverse events (irAEs) (30). In an animal study, IL-6 was found to upregulate the expression of CCR5 and arginase 1 in MDSC through a STAT3-dependent mechanism. MDSC differentiated in the presence of IL-6 strongly suppressed the function of CD8+ T cells and IL-6 overexpressing tumors grew at a significantly slower rate in CD8+ T cell-activated mice. Meanwhile, a correlation between IL-6 levels, phosphorylated STAT3 and CCR5 expression in tumor-infiltrating MDSC was confirmed in a RET transgenic melanoma mouse model (31). It has been shown that interleukin-6 (IL-6) inhibits the growth of melanocytes and early-stage melanoma cells but has no significant inhibitory effect on late-stage melanoma cells. However, the results of this MR analysis do not support a causal relationship between IL-6 and MM.

CRP has been used as an independent prognostic indicator in studies of a variety of diseases, including multiple myeloma, lymphoma, melanoma and ovarian, renal, pancreatic and gastrointestinal tumors (32). One study noted the importance of CRP as a predictor of survival in melanoma (33). In a study of 1144 melanoma patients (587 initial and 557 confirmed), CRP was found to be an independent prognostic marker in melanoma patients. The measurements of CRP should be considered for inclusion in prospective studies of prognosis in melanoma patients and clinical trials of systemic therapy for melanoma patients (21). A prospective study has shown that CRP can be used as a biomarker to assist immune checkpoint inhibitors in treatment of immune-related adverse events in melanoma patients (34). Several studies have shown that subsequent CRP levels are usually decreased in cancer patients with solid tumors and treated infections (35). Patients with advanced cancer are susceptible to a variety of infections that can negatively impact prognosis and increase mortality.

IL-6 and CRP are the two most commonly used biomarkers. Large-scale epidemiologic studies and meta-analyses have demonstrated a strong association between IL-6 or CRP and mortality outcomes from a variety of causes (including diseases such as cancer, cardiovascular disease and depression) (36–38). However, the potential mechanisms of IL-6 or CRP on MM remain to be elucidated. In past large randomized trials, higher IL-6 and CRP were associated with shorter overall survival in patients with metastatic melanoma who received ICI or chemotherapy (21). Low levels of IL6 and CRP may indicate a less inflammatory tumor microenvironment, which can enhance the effectiveness of treatment with ICIs. Monitoring these molecules in cancer patients undergoing immunotherapy may help identify those who are more likely to benefit from the treatment.

The aim of the this study was to investigate the causal relationship between IL-6 or CRP and MM using two-sample Mendelian randomization. The research showed that there was no significant causal relationship between IL-6 or CRP and MM. Besides, the four different methods of MR analysis came to consistent results.

The advantage of this study is that the selected GWAS sample size was large and two-sample MR analysis was performed, which can effectively avoid bias and reverse causality interference from observational studies. The reliability of the results was assessed by four methods: IVW, MR-Egger regression, weighted median method and weighted mode method. There are some limitations in this study without doubt. Firstly, all GWAS data were derived from European populations and the applicability to other populations still requires further research. Secondly, this study used the statistical results as a conclusion which does not apply for further in-depth research.

In conclusion, this study investigated the causal relationship between IL-6 or CRP and MM. The results showed that there was no significant causal relationship between IL-6 or CRP and MM.

## Author contributions

QW: Writing – original draft, Writing – review & editing. WZ: Data curation, Writing – original draft, Writing – review & editing. SP: Funding acquisition, Writing – original draft, Writing – review & editing.
